# Pulmonary Toxoplasmosis Diagnosed on Transbronchial Lung Biopsy in a Mechanically Ventilated Patient

**DOI:** 10.1155/2020/9710182

**Published:** 2020-02-21

**Authors:** Delyse Garg, Nikhil Madan, Omar Qaqish, Sandhya Nagarakanti, Vipul Patel

**Affiliations:** ^1^Division of Pulmonary and Critical Care Medicine, Department of Medicine, Newark Beth Israel Medical Center, Newark, NJ 07112, USA; ^2^Department of Medicine, Newark Beth Israel Medical Center, Newark, NJ 07112, USA; ^3^Division of Infectious Disease, Department of Medicine, Newark Beth Israel Medical Center, Newark, NJ 07112, USA

## Abstract

*Toxoplasma gondii* is a protozoan parasite that infects up to a third of the world's population. Infection is mainly acquired by ingestion of food or water that is contaminated with oocysts shed by cats or consuming undercooked meat containing tissue cysts. Primary infection is subclinical in immunocompetent hosts. Invasive toxoplasmosis often manifests as cerebral toxoplasmosis in immunosuppressed patients. In persons living with human immunodeficiency virus (HIV), toxoplasmosis occurs when CD4 counts are very low and is considered an acquired immunodeficiency syndrome (AIDS) defining illness. Pulmonary toxoplasmosis is rarely seen in the highly active antiretroviral therapy era. The diagnosis can be challenging due to the nonspecific nature of clinical and radiographic findings. In this report, we present a case of pulmonary toxoplasmosis in a new onset AIDS patient, which was initially clinically misdiagnosed as *Pneumocystis jiroveci* pneumonia (PJP). Due to a poor response to treatment for PJP, the patient underwent a transbronchial lung biopsy, which led to the diagnosis of pulmonary toxoplasmosis.

## 1. Introduction

The pattern of pulmonary disease in patients with HIV infection has changed with the advent of highly active antiretroviral therapy (HAART) [1]. Opportunistic infections (OI) such as *Pneumocystis jiroveci* pneumonia (PJP) caused by *Pneumocystis jiroveci* (previously called *Pneumocystis carinii*), Kaposi's sarcoma caused by human herpes virus 8 (HHV8), mycobacterial infections, and toxoplasmosis have become less prevalent [2]. However, such opportunistic infections are still diagnosed in patients unaware of their HIV status or are noncompliant with HAART [2, 3]. Although toxoplasmosis is extremely rare in the HAART era, it remains one of the most common human zoonotic diseases. About one-third of the world's population is infected with *Toxoplasma gondii*[4]. The prevalence can vary between regions, from low sero-prevalence (10–30%) in North America, Southeast Asia, and Northern Europe to high prevalence in Latin America and tropical Africa [5]. It is an intracellular parasite and can infect humans and a wide variety of mammals and birds. It was first described by Nicolle and Manceaux in 1908 [6]. The entire life cycle with the role of cats as a definitive host was understood in the 1960s [7]. The three infective stages of *T. gondii* are the invasive tachyzoite, the slow dividing bradyzoite, and the sporozoite which is protected inside an oocyst in the environment. Humans get infected by mostly consuming contaminated water and food. There is also a 30% chance of vertical transmission leading to congenital toxoplasmosis. *T. gondii* can invade a variety of host cells infecting multiple organ systems.

## 2. Case Report

**Figure 1:**
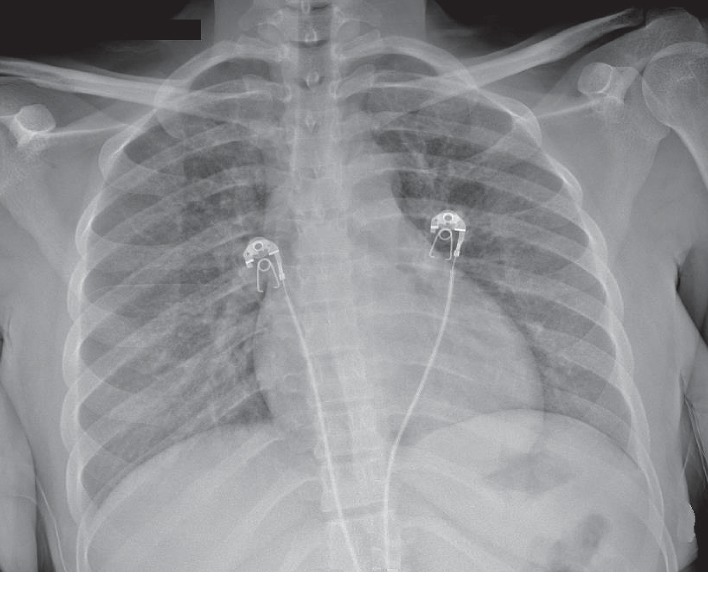
Chest radiograph showed bilateral homogeneous ground-glass opacity.

**Figure 2:**
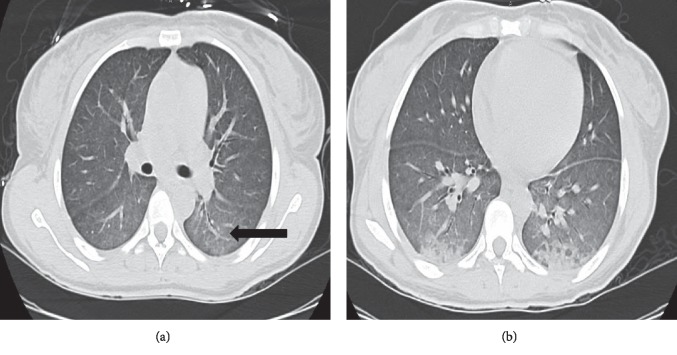
(a, b) CT scan of the chest showed lower lobe predominant confluent ground-glass opacities (arrow).

A 37-year-old woman, with no significant past medical history, presented to the hospital with acute onset of dry cough and dyspnea on exertion for a week. She did not complain of fevers or chills. The patient did not have any recent sick contacts. She did report loss of appetite and weight loss of about 20 pounds over a two-month period. She spent most of her life in Nigeria and immigrated to the United States 2 years ago, working as a cultural activist in her community. She was married with one child who lives in her home country along with her spouse. She lived with friends and had no pets. She was a nonsmoker with no alcohol or illicit drug use. On admission, the patient was in mild respiratory distress. Her vitals showed a heart rate of 152 beats/minute, blood pressure of 116/83 mmHg, respiratory rate of 22 breaths/min, temperature of 99°F, and SaO_2_ of 96%, while breathing ambient air. Physical examination revealed hepatomegaly, but the other systems exam was normal. Laboratory examination revealed hemoglobin (Hgb) of 11.8 grams/deciliter, platelet (PLT) count of 94,000 cells/mcl, aspartate aminotransferase (AST) of 664 units/liter (L), and alanine aminotransferase (ALT) of 1703 units/L. Lactate dehydrogenase (LDH) was elevated at 3462 units/L, and creatinine phosphokinase (CPK) was also elevated at 2742 units/L. A rapid HIV test was positive, and the CD4 count was 8 cells/mcl with HIV viral load of greater than 10 million copies/milliliter (mL). Serological tests revealed active hepatitis B infection with a positive hepatitis B surface antigen and hepatitis B core total antibody and a negative hepatitis B core IgM and hepatitis B surface antibody. Hepatitis B viral load was greater than 17 million DNA units/L. Chest radiograph showed bilateral homogeneous ground-glass opacity (Figure 1). A computed tomography (CT) scan of the chest showed lower lobe predominant bilateral ground-glass opacities and bilateral lower lobe atelectesis (Figures 2(a) and 2(b)). Differential diagnosis included, but was not limited to PJP, atypical pneumonia and viral infections. The patient was started on empiric treatment for PJP with intravenous sulfamethoxazole-trimethoprim at 220 mg (trimethoprim component) every 6 hours along with systemic corticosteroids (oral prednisone 40 milligrams/day). She was also started on treatment for community-acquired pneumonia with intravenous ceftriaxone 2 grams and azithromycin 500 mg daily, while awaiting culture results.

**Figure 3:**
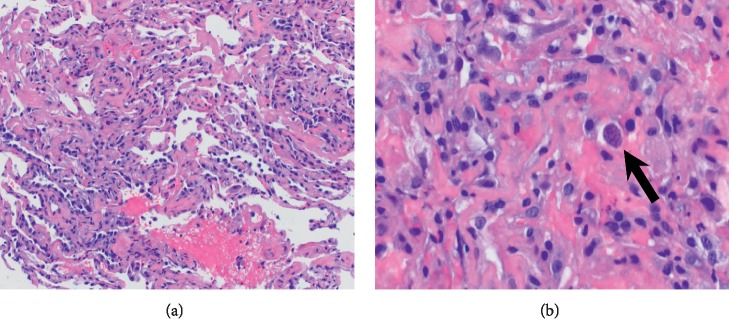
(a, b) Hematoxylin and eosin stain showed thickening of respiratory membrane and increase type II pneumatocytes. High-power view showed a cyst containing discrete granule-type bradyzoites in lung parenchyma (arrow).

**Figure 4:**
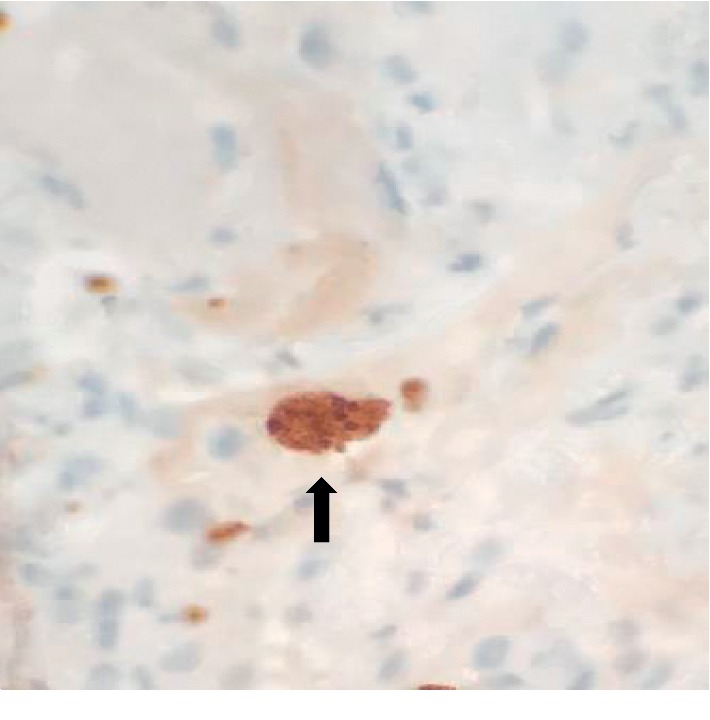
Toxoplasma immunohistochemical stain showed cyst-containing bradyzoites (arrow).

Over the next 48 hours, respiratory distress worsened, requiring mechanical ventilation. Arterial blood gas analysis revealed arterial oxygenation of 157 millimeters of mercury (mm Hg) on a 100% inhaled oxygen with an A-a gradient of 506 mm Hg. She underwent flexible bronchoscopy with bronchoalveolar lavage (BAL) and transbronchial lung biopsy (TBLB) due to poor response to standard therapy for PJP. BAL showed many alveolar macrophages with no evidence of *Pneumocystis jiroveci* or any other organisms. Hematoxylin and eosin stain of lung biopsy showed a toxoplasma cyst containing bradyzoites in the lung parenchyma (Figures 3(a) and 3(b)). Immunohistochemical stain directed for *Toxoplasma gondii* confirmed the diagnosis (Figure 4). Her serum *Toxoplasma* IgG was positive, but IgM was negative. Subsequently, appropriate treatment with oral sulfadiazine 1 gram every 6 hours and pyrimethamine 50 mg per day along with folinic acid 25 mg per day was initiated. Antibiotics for community-acquired pneumonia were discontinued after careful review of all cultures. The patient was liberated from mechanical ventilation within 72 hours. Prednisone was discontinued as its role in pulmonary toxoplasmosis has not been well studied.

**Figure 5:**
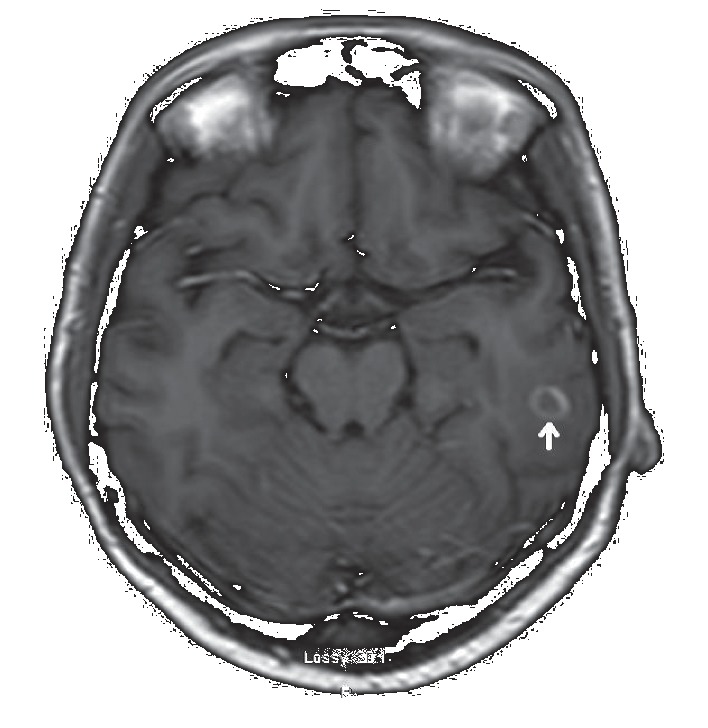
MRI brain showed a peripheral lesion with ring enhancement measuring 1 cm (arrow) in the posterior left temporal lobe with surrounding vasogenic edema without significant mass effect in the ipsilateral basal cistern.

Though the patient did not have any neurological symptoms or signs throughout the course of hospital admission, a decision was made to obtain magnetic resonance imaging (MRI) of the brain. MRI showed a lesion in the left temporal lobe with surrounding edema (ring enhancing lesion) measuring 1 cm (Figure 5). A prolonged course of pyrimethamine 1 gram every 6 hours and sulfadiazine 50 mg/day along with folinic acid 25 mg/day was continued with close monitoring of the brain lesion. During her hospital course, liver enzymes showed improvement. On discharge from hospital, AST was 98 units/L and ALT was 105 units/L. Antiretroviral therapy was not initiated in the hospital due to concern of the possibility of causing an immune reconstitution syndrome. The patient was discharged from the hospital in a stable condition. She will follow up in an infectious disease clinic with plans to start treatment for HIV and hepatitis B infections. Hepatitis B therapy was planned to be given as an outpatient along with HIV therapy by choosing HAART regimens which have activity against hepatitis B infection as well.

## 3. Discussion

Primary infection with toxoplasma in immunocompetent hosts usually goes unnoticed in about 80% of the individuals but can present with flu-like symptoms, asthenia, lymphadenopathy, and hepatosplenomegaly [8]. Lymphadenopathy and asthenia can persist for several weeks. On the contrary, infection in immunocompromised hosts is always life threatening due to significant impairment in cellular immunity. Patients usually have a reactivation as a result of cyst rupture than a newly acquire infection. In transplant patients, toxoplasmosis can occur as a result of reactivation of latent infection in the recipient or infection from a cyst-containing organ donor. HIV-related pulmonary toxoplasmosis was first described in 1984 [9]. It is encountered in association with CD4 counts of less than 100 cells/mcl and is therefore considered to be an AIDS defining illness [10]. In HIV-infected patients, it usually manifests as central nervous system disease in the form of focal brain lesions [11]. Toxoplasmic encephalitis can present with headaches, lethargy, incoordination, ataxia, and seizures usually associated with fevers [12]. Extra cerebral toxoplasmosis most commonly affects the eye, followed by the lungs and heart [13]. It can also affect other organ systems such as liver, pancreas, bone marrow, lymph nodes, kidney, spleen, and skin. Respiratory manifestations of acute toxoplasmosis are usually described in transplant recipients and patients with HIV infection [10, 14, 15]. Pulmonary toxoplasmosis accounted for 4% of all cases of pneumonia in the HIV-infected patients in the pre-highly active antiretroviral therapy (HAART) era and is uncommon in the HAART era [9, 10]. Pulmonary toxoplasmosis commonly presents as a subacute febrile illness with cough and dyspnea [10, 16]. Radiographic abnormalities predominantly comprise two patterns: bilateral, diffuse, fine to medium reticulonodular opacities, such as those seen in PJP, and a bilateral, predominantly coarse, nodular pattern more commonly seen in tuberculosis, histoplasmosis, and coccidioidomycosis [15–17]. Given the nonspecific nature of symptoms and radiological tests, and the significant overlap with other etiologies, the diagnosis of pulmonary toxoplasmosis can be challenging. Given the aggressive nature of the disease in immunocompromised patients, it is a diagnostic emergency.

Direct examination of the Giemsa-stained body fluid, mainly cerebrospinal fluid (CSF), bronchoalveolar lavage (BAL), or tissue sections with a demonstration of the parasite is the fastest means of diagnosis, but it lacks sensitivity. Detection of parasite DNA by real-time polymerase chain reaction (PCR) in the body fluid has emerged as one of the most useful diagnostic tools in the immunocompromised population. The *Toxoplasma* PCR assays target several genes and loci, most commonly the 35-repeat B1 gene and single-copy gene (P30), which codes for the major surface antigen P30. Peterson et al. studied the utility of real-time PCR in BAL samples from 332 HIV-infected patients with pulmonary infections of unknown etiology. Positive results were seen in 7 patients, and they concluded that PCR for detecting *T. gondii* in BAL samples should be performed in all immunosuppressed HIV-positive patients with suspected infection of unknown etiology [18]. Sensitivities of PCR in CSF and blood in HIV-infected patients were about 69 to 83% [19] and 80% [20], respectively.

Serological testing is the mainstay of diagnosis in immunocompetent hosts. Immunoglobulin (Ig) A and IgM antibodies are produced during the first week after infection and reach a plateau within 1 month. IgM antibodies start to decline after that and disappear around 7 months to a year [21]. IgG can be detected 1–3 weeks after IgM, and it plateaus around 2–3 months and remains positive for life. Serology in immunocompromised patients has limited utility with the majority showing IgG positivity, some are positive for IgG and IgM while others are negative for both IgG and IgM [13]. Serologic testing is useful when it is negative for a patient with symptoms of acute toxoplasmosis. It can also be used to monitor titers and prompt further testing in cases of strong increases in IgG titers [22]. It is possible to see the reappearance of IgM antibodies in reactivation of the disease, especially in transplant recipients. In HIV-infected patients, rise in IgG titers can correlate with the occurrence of cerebral toxoplasmosis; however, the time line is not well defined [23]. Our patient likely had reactivation of prior toxoplasma infection as she had positive IgG and negative IgM toxoplasma serology.

If BAL is nondiagnostic, then a transbronchial or open lung biopsy (OLB) should be pursued. Diagnostic yields for these procedures in pulmonary toxoplasmosis in HIV-infected patients have not been well studied. Transbronchial lung biopsy (TBLB) is considered relatively unsafe while on mechanical ventilation due to increased risk of complications, especially pneumothorax. Preliminary data have suggested that TBLB may be safer than open lung biopsy (OLB) with fewer complications [24, 25]. There are limited, but encouraging, data showing that TBLB can be performed with an acceptable risk and have a significant diagnostic yield in mechanically ventilated patients. A definitive diagnosis is made in 35–74% of mechanically ventilated patients via TBLB, resulting in a change in management in 41–63% patients based on biopsy findings [26, 27]. Although complications can occur, the benefits appear to exceed the risks in patients in whom a histological diagnosis is necessary, often obviating the need to perform a surgical lung biopsy. Asai et al. noticed no significant difference in the rate of complications between mechanically and nonmechanically ventilated patient groups [28]. OLB is reserved for patients without a diagnosis after extensive testing. It has very high diagnostic yield as seen in a study by Miller et al., where an open lung biopsy provided a diagnosis in all 23 HIV-infected patients with acute respiratory episodes and 21 of those had undergone BAL with or without TBLB [29]. In another study, OLB provided a diagnosis in all 26 HIV-infected patients with unexplained pulmonary infiltrates in the HAART era. There was a 27% in-hospital mortality [30].

Toxoplasmosis in the immunocompromised host can cause significant morbidity and mortality if not diagnosed and treated early [10, 11, 13]. In our patient, treatment for PJP was initiated empirically based on radiological findings, which has significant overlap with treatment of toxoplasmosis; however, the duration of treatment is different in each infection. The detection of pulmonary infection also helped us to detect cerebral toxoplasmosis. This case also highlights the importance of utilizing bronchoscopy with TBLB in patients with radiologic findings with unclear diagnosis in patients with respiratory failure requiring mechanical ventilation, which was not reported in previous pulmonary toxoplasmosis. A significant delay in diagnosis was avoided by obtaining a histological examination of tissue early.

## 4. Conclusion

Toxoplasma pneumonia should be considered in the differential diagnosis of respiratory illness with bilateral infiltrates on chest imaging, especially in immunocompromised hosts as the presentation is nonspecific. Pulmonary toxoplasmosis can mimic PJP, and it should be suspected when there is inadequate response to trimethoprim-sulfamethoxazole in suspected PJP to avoid sudden worsening of the clinical condition. Early diagnosis and prompt treatment are crucial to prevent the significant morbidity and mortality associated with this entity. TBLB should be considered in patients with pulmonary infiltrates with unclear diagnosis.
